# The Treatment of Impaired Wound Healing in Diabetes: Looking among Old Drugs

**DOI:** 10.3390/ph13040060

**Published:** 2020-04-01

**Authors:** Simona Federica Spampinato, Grazia Ilaria Caruso, Rocco De Pasquale, Maria Angela Sortino, Sara Merlo

**Affiliations:** 1 Department of Biomedical and Biotechnological Sciences, Section of Pharmacology University of Catania, 95123 Catania, Italy; simona_spampinato@hotmail.com (S.F.S.); grazia.caruso@outlook.it (G.I.C.); sara_merlo@hotmail.com (S.M.); 2 Ph.D. Program in Biotechnologies, Department of Biomedical and Biotechnological Sciences, University of Catania, 95123 Catania, Italy; 3 Department of General Surgery and Medical-Surgical Specialties, University of Catania, 95123 Catania, Italy; r.depasquale@unict.it

**Keywords:** wound closure, diabetic foot ulcer, DPP4 inhibitor, metformin, phenytoin, statins, drug repurposing

## Abstract

Chronic wounds often occur in patients with diabetes mellitus due to the impairment of wound healing. This has negative consequences for both the patient and the medical system and considering the growing prevalence of diabetes, it will be a significant medical, social, and economic burden in the near future. Hence, the need for therapeutic alternatives to the current available treatments that, although various, do not guarantee a rapid and definite reparative process, appears necessary. We here analyzed current treatments for wound healing, but mainly focused the attention on few classes of drugs that are already in the market with different indications, but that have shown in preclinical and few clinical trials the potentiality to be used in the treatment of impaired wound healing. In particular, repurposing of the antiglycemic agents dipeptidylpeptidase 4 (DPP4) inhibitors and metformin, but also, statins and phenyotin have been analyzed. All show encouraging results in the treatment of chronic wounds, but additional, well designed studies are needed to allow these drugs access to the clinics in the therapy of impaired wound healing.

## 1. Introduction

Diabetes mellitus (DM) is defined as a group of metabolic pathologies characterized by compromised insulin production and/or function, leading to hyperglycemia. DM greatly impacts the quality of life and survival expectation of patients. In fact, as a consequence of hyperglycemia, diabetic patients are at increased risk for comorbid conditions affecting several organs [1]. One of the main consequences of diabetes is the impairment of self-repairing abilities [2].

## 2. The Problem of Wound Healing and Its Incidence in Diabetes

Impaired healing in diabetes is the result of a complex pathophysiology involving vascular, neuropathic, immune, and biochemical components [3]. Hyperglycemia correlates with stiffer blood vessels which cause slower circulation and microvascular dysfunction, causing reduced tissue oxygenation [2]. Blood vessel alterations observed in diabetic patients also account for reduced leukocyte migration into the wound, which becomes more vulnerable to infections [3]. The hyperglycemic environment itself can compromise leucocyte function. In addition, peripheral neuropathy can lead to numbness of the area and reduced ability to feel pain, which can lead to chronicization of wounds that are not immediately noticed and properly treated [3]. The described features are particularly relevant in the lower limbs and particularly the foot, more exposed to even minor wounds and thus more susceptible to chronicization. In addition, alterations of motor and sympathetic functions lead to physical deformation of the foot and increased plantar pressure, as well as excessive skin dryness which can further favor cracks and unnoticed small wounds [3].

Diabetic patients have a 15–25% lifetime risk of developing diabetic foot ulcers, of which 40–80% become so severely infected it involves the bone, leading to osteomyelitis [4]. Global epidemiological studies suggest a higher prevalence in North America [5]. A high number of cases of foot ulcerations require hospitalization and surgical intervention with amputation of the interested body part. In addition, the rate of recurrence of a foot ulcer is greater than 50% after 3 years from the first episode [6]. For this reason, impaired wound healing in DM represents a major healthcare issue and a significant economic burden [7,8]. Notably, costs for diabetic foot ulcerations treatment are additive with general costs for diabetes care, and are on the rise with the increased incidence and prevalence of the pathology [6,7,9]. 

## 3. The Physiology of Wound Healing

Wound healing is a physiologic, very complex phenomenon that occurs when skin integrity is lost and consequently also the barrier function of the skin is impaired. This may occur quite often since the skin is much exposed to external insults and the need to avoid systemic infections drives a rapid defense mechanism [10]. Through physiologic healing, the normal status of the skin can be fully recovered although only a maximum of 70% of previous tensile strength is usually achieved [11]. This process progresses in different subsequent steps and usually three successive phases are recognized: an inflammatory, a proliferative, and a remodeling phase.

### 3.1. The First Phase: Inflammatory Response

The inflammatory phase is preceded by a coagulation step in which the first response of the injured skin is activation of the clotting cascade with recruitment of platelets and the formation of a fibrin plug. Its significance resides in hemostasis as well as wound coverage and protection [12]. Aggregated platelets within the clot form also the basic structure for the recruitment of inflammatory cells and, through the release of several cytokines and growth factors, including platelet-derived growth factor (PDGF) and transforming growth factor β (TGFβ), they attract different cell types. These include neutrophils and monocytes whose passage is favored by concomitant vasodilation. The latter convert into macrophages very early that, again through the release of growth factors and cytokines, are considered the main cellular actors of this inflammatory phase. Keratinocytes migrate in the wounded area as well and local fibroblasts start proliferating [13]. These events occur early, starting a few hours after injury and proceed fast so that by 48–72 h all the different recruited cell types contribute to the formation of the early granulation tissue [14].

### 3.2. The Second and Third Phases: Proliferation and Remodeling

These phases are aimed at driving wound closure. In this regard, granulation tissue, together with keratinocyte migration, formation of extracellular matrix (ECM) proteins, and the appearance of myofibroblasts cause contraction, one of the first events leading to wound closure. Fibroblasts are then the main cell type in this phase and, through the release of collagen, they start to rebuild the wounded area. The early phases of wound healing are characterized by hypoxia that plays a role in promoting migration and proliferation of each cell type as well as release of growth factors [14]. Hypoxia induces in fact activation of hypoxia inducible factor (HIF)-1α and stimulation of vascular endothelial growth factor (VEGF-A), one of HIF-1α principal target genes, is induced [15]. As a consequence, active proliferation of endothelial cells occurs and this is at the basis of neo-angiogenesis, critical for vital support to the forming tissue. VEGF-A causes increased capillary density in the wound and restoration of blood flow that promotes wound healing [16]. This initial proliferative phase, characterized by chaotic and abundant vessels formation, is followed by a maturation phase in which, through the recruitment of pericytes that release antiangiogenic and vascular maturation factors, selected vessels become durable and mature [17]. Hence healing evolves towards the remodeling step in which collagen type III, which first accumulates, is replaced by type I (as usually observed in normal, non-injured tissue) [18], neovascularization is contained and goes back to normal [19], and the wound repair process proceeds towards restoration of the physiologic structure of the skin [20]. 

When the physiologic reparative process fails, a chronic wound condition takes place with the appearance of an ulcer or, alternatively, an excessive repair event with the formation of a hypertrophic scar or a keloid. The appearance of hypertrophic scars usually associates with patient’s predisposition, they are temporary and usually regress within six months. In contrast, keloids cover an area that goes beyond the original skin injury, appear in genetically predisposed patients and are permanent [10]. On the other side, ulcers often occur in ischemic conditions, but are typical of diabetic patients, representing one of the major delayed complications of the disease.

## 4. What Goes Wrong in Wound Repair in Diabetes?

The healing process in diabetes is mainly characterized by chronicization of the inflammatory conditions, disrupted angiogenic process, reduction of endothelial progenitor cells, and an imbalance in extracellular matrix regulation. As observed in physiological wound repair, also in diabetes, neutrophils and macrophages promptly infiltrate the area of the lesion driven by chemotactic chemokines that are particularly elevated in diabetes [21]. Infiltrating cells release inflammatory cytokines such as interleukin 1β (IL-1β) and tumor necrosis factor α (TNFα) whose levels are elevated not only during the initial inflammatory acute repair phase, but remain at high concentrations in the wounded area for longer time, thus indicating the maintenance of a prolonged inflammatory response [21]. In diabetes the production of several growth factors involved in initiating and sustaining the healing process is compromised. For instance, reduced levels of insulin like growth factor-1 (IGF-1) and transforming growth factor-β (TGFβ) have been reported in the wound tissue in both diabetic animals and humans. IGF-1 is implied in cell-granulation and wound re-epithelization [22], while TGFβ recruits immune cells, keratinocytes, fibroblast, and vascular cells and is involved in angiogenesis and formation of the ECM [23]. However, in diabetes, the balance between the promotion of new vessels formation and their maturation is perturbed. Angiogenesis is dysfunctional in endothelial cells exposed to high glucose levels and in the wound area capillary density is insufficient. Hyperglycemia affects HIF-1α stability and activation and consequently it suppresses HIF-1α target genes like VEGF [24]. Furthermore, in diabetes animal models, macrophages, which are the main source of VEGF, exhibit impaired phagocytic activity and altered phenotype, resulting in failure of tissue repair [25]. Accordingly, in db/db mice VEGF-A mRNA and protein levels were significantly reduced compared to control mice [26] and treatment with VEGF-A caused accelerated wound closure, although this was characterized by early leaky and malformed vasculature and a big local edema very evident until VEGF-A treatment was ceased [27]. In this context, dysregulated production of both pro-angiogenic and vascular maturation factors, observed in diabetes, leads to a reduced population of endothelial progenitor cells in the bone marrow [28], thus causing modifications in angiogenic sprout and, in conclusion, an aberrant vascular architecture in diabetic wounds [29].

Additionally, the maturation phase of wound healing appears impaired in diabetes. The production of factors leading to vascular mature phenotype (including angiopoietin (ANG) 1 and 2, PDGF) is compromised [30] and topical application of ANG1 and PDGF increased wound healing in a mouse model of diabetes induced by streptozotocin or in db/db mice, respectively [30,31].

Finally, an impairment in the regulation of ECM, whose build-up is modulated by metalloproteinase (MMPs) and tissue inhibitors of metalloproteinase (TIMPs) is observed in diabetes. Higher MMPs levels have been reported in diabetic wounds, due to high glucose that may directly induce the production of MMPs and the reduction of TIMPs, thus contributing to disruption of the healing process [32]. 

**Figure 1:**
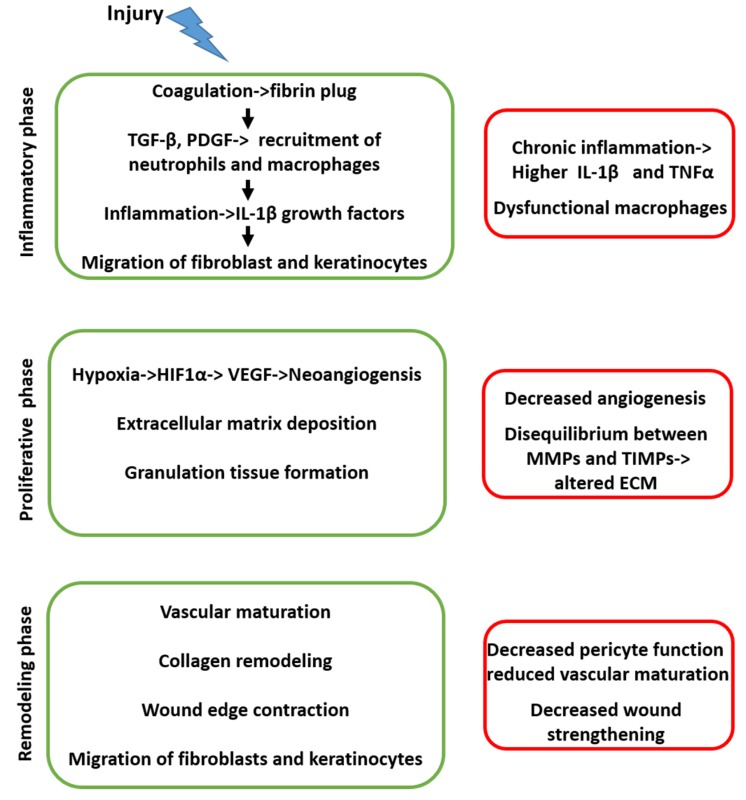
The series of events that occur in sequence during physiological (left side, green) and diabetic (right side, red) wound healing.

MMPs are involved at various stages of wound healing, like cell migration through the degraded ECM, leukocyte invasion, processing of multiple cytokines, and growth factors involved in the healing process [33]. The balance between MMPs and TIMPs is essential to avoid the disruption of the scaffolding structures necessary for a proper wound healing [33]. A synthetic, schematic picture of the sequence of events in physiological and diabetic conditions is reported in Figure 1. 

## 5. Current Available Treatments

The standard wound-care practice for impaired wound healing includes control of the infection together with debridement, off-loading to relieve pressure, and maintenance of a moist wound bed. 

One of the first aims in the treatment of chronic wounds is in fact to prevent the occurrence of infection and to clean the area from non-viable tissue material. Infections can easily occur due to the loss of the innate barrier constituted by the skin layer and pathogens accumulating in the wounded area may further interfere with the healing process. Similarly, the removal of debrides and non-viable tissue, or debridement, allows exposure of healthy tissue where cells can migrate and proliferate to repair the wounded area [20]. There are several debridement methods including the enzymatic, based on the self-activation of endogenous enzymes involved in fibrin degradation, but also mechanical and surgical. The latter appears the most efficacious resulting in complete removal of the necrotic tissue and prevention of its spreading out.

### 5.1. Dressings

Following these initial phases, current treatment of impaired wound healing relies mainly on the use of topical dressings that, by providing the moist environment required for proper healing, facilitate the production of granulation tissue and epithelialization [34]. They also contribute to decrease the risk of infection, accelerate wound healing, and reduce scarring. Different kinds of dressings have been developed, and all of them share the above described features: promotion of reepithelization, moisture, antimicrobial properties, prevention of further trauma, exudate wicking [35]. They can be either non-medicated or medicated, passive, bioactive, and interactive. Passive dressings are used only to cover the wound and to allow healing. Interactive dressings constitute a barrier against the access of bacteria. They can be occlusive or semi-occlusive and made of different materials. Among others, films, foams, alginates, hydrogels, and hydrocolloids are some of the more commonly utilized [36]. Specific material may be preferred to the others depending on the parameters of the wound. In the bioactive dressing, a moist environment and the presence of growth factors promote the formation of the granulation tissue. They are produced from biomaterials such as collagen, hyaluronic acid, chitosan, and often growth factors and antimicrobials are added to enhance the wound healing property [36].

### 5.2. Antidiabetic Drugs

Interestingly, drugs that are commonly used in the therapy of diabetes including insulin, metformin, some sulfonylureas, thiazolidinediones, and DPP-4 inhibitors have shown not only anti-inflammatory properties, but also a broad range of different effects that may be useful in the treatment of chronic wounds. In detail, specific beneficial effects of these medications such as polarization towards a healing-promoting macrophage phenotype, reduction of MMPs, increased keratinocyte and fibroblast proliferation, angiogenesis, and increased formation of granulation tissue have been reported. However, whether this correlates clinically with improved wound healing, in some cases, has to be demonstrated [37]. In selected examples, as detailed below, data are promising and deserve the right attention and more focused studies. 

### 5.3. Growth Factors

Treatment of chronic wounds with exogenous growth factors has gone through various and contrasting phases. Several growth factors bear the potential to intervene at various stages of the healing process activating a variety of cellular and molecular responses [38]. They can in fact stimulate the formation of granulation tissue, modulate the inflammatory response, promote angiogenesis, stimulate ECM formation, remodeling, and also re-epithelization. Some of them including PDGF, VEGF, EGF, FGF, and TGFβ1 have also been tested in clinical trials, more specifically for the treatment of diabetic foot ulcers. Although encouraging, results of these studies revealed a high risk of bias and safety issues were not well addressed [39]. Worth mentioning is the availability of a topical formulation of recombinant human PDGF-BB in the market for over 10 years in Europe and still present in the United States, marketed as an adjuvant therapy for diabetic neuropathic ulcers [40]. The safety issue of this product is somehow debatable and great caution is requested in patients with neoplastic diseases. One of the main limitations of the incomplete success of the growth factor preparations may be linked to their unsatisfactory formulations, for example of PDGF or EGF, so that innovative drug delivery systems have been proposed [41]. Today, the controlled release of growth factors is guaranteed by polymeric micro- and nanospheres, lipid nanoparticles, hydrogels, scaffolds, nanofibrous structures that can increase the stability of the protein at the wound site, allowing optimization of the treatment [41]. A more recent approach points to degradable biomaterials carrying the growth factors or, alternatively, a gene-mediated therapeutic delivery to yield high concentration of the growth factor(s) selectively in the wounded area [42]. 

### 5.4. Stem Cells

The possibility of applying stem cell therapy in the treatment of impaired wound healing appears rather intriguing. Transplanted stem cells can in fact release, in the wounded area, cytokines and growth factors able to promote cell recruitment, angiogenesis and ECM remodeling and exert an immunomodulatory action [43,44]. Adult mesenchymal stem cells (MSC) have shown efficacy in several clinical trials and are included in commercially available topical products [45,46]. More recently, the use of induced pluripotent stem cells (iPSC) has emerged as a novel cellular therapy with the advantage of being potentially an autologous transplant with low rate of immune rejection. A large body of data is provided by several preclinical studies carried out in animal models of wound healing [47] that make promising the opportunity to translate iPSC into a new therapeutic tool for wound healing also in humans in the near future. 

## 6. Drug Repurposing in The Treatment of Wound Healing

From that described above, there are several different therapeutic approaches that are available, but all of them carry limitations and none appear adequate to guarantee a successful, conclusive, non-recurrent healing. Hence, the need to develop (or to find) new treatment alternatives is mandatory. Since the sequence of events of impaired wound healing is well characterized, the possibility to identify drugs that are already in the market and that are known to target one or, even better, many of the described molecular events can be taken into account. In this regard, several molecules with different therapeutic indications have shown beneficial effects in promoting wound healing. Sometimes observations have been occasional as in the case of the highest incidence of chronic non-healing wounds in male patients suggesting that the female hormone, estrogen, can be beneficial in the treatment of chronic wounds. Accordingly, topical estrogens accelerate wound healing, favor the formation of capillary-like structures in endothelial cells, stimulate the release of PDGF by macrophages and TGFβ1 by fibroblasts and promote wound contraction, formation of granulation tissue and collagen deposition [48]. More recent data indicate that estrogens enhance wound closure and reepithelization also in diabetic mice and this effect is mediated by increased epithelial precursor cells and mesenchymal stem cells which contribute to neo-angiogenesis and tissue regeneration, respectively [49]. Interestingly, the migratory promoting effect of keratinocytes that can produce re-epithelization seems to involve mainly estrogen receptor β (ERβ) [50] and accordingly genistein, an isoflavone acting selectively on ERβ, displays anti-inflammatory and antioxidant activity while improving wound healing in diabetic mice [51]. Though promising, a gap still exists between this large body of evidence and the possibility to translate it to the clinic.

Despite controversial results are available regarding the use of systemic propranolol in the treatment of chronic wounds, this β-blocker has been tested since activation of β2-adrenergic receptor is known to inhibit keratinocyte migration and delay re-epithelialization. Interesting data were provided by a study in which a 1% propranolol cream was applied to chronic wounds in spontaneously diabetic mice [52]. Topical propranolol was effective in inducing re-epithelialization, functional angiogenesis, and increased ECM turnover confirming its potential in the treatment of wound healing.

The angiotensin converting enzyme (ACE) inhibitor, captopril, has shown the ability to increase wound healing scores in diabetic rats [53] and its recognized activity as a reactive species scavenger has prompted its inclusion in novel wound dressings together with biologically derived materials. This combination results in a better wound reparative activity compared to the biomaterial applied alone [54].

Besides those mentioned above, there seems to be several other currently used drugs that have shown some activities in the wound repair process. However, we will here selectively focus on few classes of drugs (including some antidiabetic medications) whose potential to be effective in the treatment of wound healing finds support in preclinical and clinical observation and is also substantiated by detailed molecular evidence. They are all small molecules and their chemical formulas and structures are reported in Table 1.

The chemical formula and structure of lovastatin, the first statin to reach the market, is shown. Other available statins include atorvastatin, fluvastatin, pravastatin, simvastatin, rosuvastatin, and pitavastatin. The chemical formula and structure of sitagliptin, the first DPP4 inhibitor to reach the market, is shown. Other available DPP4 inhibitors include vildagliptin, saxagliptin, linagliptin, gemigliptin, anagliptin, teneligliptin, alogliptin, trelagliptin, and omarigliptin 

### 6.1. Statins

Statins are 3-hydroxy-3-methylglutaryl-coenzyme A (HMG-CoA) reductase competitive inhibitors and represent one of the first line classes among cholesterol-lowering drugs. Inhibition of cholesterol production by statins implies also inhibition of the synthesis of the wound-healing inhibitors farnesyl pyrophosphate (FPP) and cortisol. They both act on glucocorticoid receptors and inhibit epithelization through impairment of keratinocyte migration. Statins are also known to exert cholesterol-independent pleiotropic effects and are endowed with anti-inflammatory, antibacterial, and antioxidant properties, as well as the ability to ameliorate endothelial function and healing outcomes [55].

Simvastatin has been shown to improve tissue repair by promoting neovascularization, mobilization of endothelial precursor cells from bone marrow, and increase of their proliferation, migration, and survival [56]. Increased VEGF and NO content in the wound area may account for the ameliorated healing capacity in diabetic animal models [57]. It also exerts anti-inflammatory and antibacterial activity in a variety of wound conditions including impaired diabetic healing in animal models [58]. Furthermore, simvastatin shows an antiapoptotic effect on lymphatic endothelial cells, thus promoting lymphangiogenesis, particularly favorable in subjects with defective microcirculation, such as diabetic patients [59]. Interestingly, different statins including mevastatin, atorvastatin and pravastatin share the same ameliorating effect on wound healing. Some mechanistic investigations at cellular level have shown that mevastatin switches human keratinocytes from a hyperproliferative to a promigratory phenotype, by modulating EGF signaling and stimulating cytoskeletal reorganization and lamellipodia formation. These effects were observed also in biopsies from diabetic foot ulcer patients strengthening the possible use of a topical statin in the treatment of diabetic ulcers [60]. In a porcine wound model, topically applied mevastatin improved epithelization and angiogenesis by contrasting cortisol and FPP actions. All these effects likely involved induction of a long non-coding RNA, lnc-RNA Gas5, known to regulate cell proliferation and viability [61]. Due to the low concentrations of statins yielded at the skin, more recent research pointed to particular formulations of statins aimed at optimizing drug delivery at the wounded area. Hence, simvastatin has been formulated for intraosseus injection and administered to streptozotocin-induced type 1 diabetic rats carrying a full-thickness dermal wound. A single injection of simvastatin ameliorated wound healing while promoting endothelial progenitor cell mobilization and neovascularization [62]. Moreover, application of a lovastatin-loaded tissue engineering scaffold, which offers the advantage of mimicking ECM with integrated drug delivery capacity, was able to increase wound healing rate by enhancing eNOS and skin blood flow within and around the wounded skin [63].

### 6.2. Phenytoin

Gingival fibrous overgrowth is a common side effect of phenytoin when the drug is used as an anticonvulsant and occurs in about 50% of treated patients. The mechanism of action of phenytoin in the control of seizure involves blockade of voltage-dependent Na+ channels but little is known on the molecular process that leads to hypertrophy of the gum. The gingival hypertrophy is so evident that it prompted direct testing of phenytoin first in experimentally induced gingival wounds to move quickly to skin reparative processes, as recently reviewed [64], with limited investigation on the mechanistic events at the basis of the observed effects. Hence a detailed description of the molecular mechanism of action induced by phenytoin at the skin is limited and most literature on this topic dates back to more than two decades ago [65]. Phenytoin increases selectively the proliferation of fibroblasts, but not of other cell types, in vitro [66,67], but an indirect control of fibroblast proliferation through the release of cytokines from keratinocytes has also been suggested [68]. Phenytoin stimulates the formation of granulation tissue, reduces collagenase activity, and promotes collagen production and deposition thus causing enhanced strength of the wounded area [69]. It also exerts antibacterial activity by reducing the bacterial load of the wound. Whether this effect is direct or secondary to resolution of the inflammatory response with reduction of edema and exudate has to be established [65]. Phenytoin has also shown the ability to increase VEGF and FGF at the wound site with a consequent stimulation of new vessel formation [70]. Accordingly, biopsies of wound tissue treated with phenytoin exhibit signs of increased collagenization, neovascularization, and reduced infiltration of circulating inflammatory cells.

A topical formulation of phenytoin was then formulated and proved to be efficacious in accelerating wound healing in trophic leprosy ulcers [71] and post melanocytic nevi surgery [70]. Several other studies followed, either vs. placebo or other comparators, randomized or not, but most of them shared a poor description of details of the study. A systematic review has underlined this limitation, highlighting the poor methodological quality of the available studies that, however, seem to indicate a positive effect of topical phenytoin in wound healing in a large range of concentrations used, without significant systemic side effects [64]. 

### 6.3. Metformin

The biguanide metformin is recognized worldwide as the first-line drug for the treatment of type II DM. It exerts its antihyperglycemic effect at several levels including inhibition of hepatic glucose production, reduction of intestinal glucose absorption, improvement of glucose uptake, and utilization by peripheral tissues [72]. Emerging evidence indicates novel pleiotropic effects of metformin that include potential renal and cardiovascular protection, antioxidant, antifibrotic, and antiproliferative properties [73] and the ability to increase number and function of endothelial precursor cells [74,75]. Metformin’s mechanism of action is linked to inhibition of the complex 1 of mitochondrial electron transport chain and subsequent reduced ATP production and increased AMP:ATP ratio with activation of 5′-AMP-activated protein kinase (AMPK). AMPK then orchestrates all the metabolic responses of the cell, including oxidation of substrates in mitochondria and reduction of the glycolytic pathway, that are responsible for the reported effects [72,76]. In an AMPK-dependent and -independent manner, metformin downregulates mammalian target of rapamycin (mTOR) signaling, involved in the immunomodulatory effect [77]. Metformin has been shown to also modulate other pathways of inflammation such as nuclear factor kappa B (NF-kB) and mitogen-activated protein kinase (MAPK)/c-Jun NH2-terminal kinase (JNK) [78]. All this demonstrated that immunomodulatory and anti-inflammatory properties may support the potential of metformin for the treatment of wound healing. Activation of AMPK though inhibition of mTOR and NOD-like receptor protein 3 (NLRP3) inflammasome is in fact able to modulate polarization of macrophages towards the M2 phenotype, all effects that converge towards the resolution of wound closure [79]. Accordingly, topically applied metformin in a pluronic gel formulation accelerated healing of excisional wounds in rat skin with a parallel increased polarization of M2 macrophages through activation of AMPK and ensuing downregulation of the mTOR/NLRP3 inflammasome signaling pathway [80]. Faster wound healing and increased angiogenesis were observed in db/db diabetic rats following systemic administration of metformin for 14 days [81]. These effects were correlated with increased function of endothelial precursor cells and nitric oxide (NO) levels as well as antioxidant activity. Metformin was also able to restore basal levels of thrombospondin 1, an endogenous antiangiogenic mediator known to be involved in vascular complications in diabetes [81]. Notably, metformin was effective in accelerating wound healing by improving epidermis, hair follicles, and collagen deposition also when applied topically in young rats undergoing an excision wound [82,83] and confirmed its efficacy also in patients carrying non-healing lower limb traumatic wounds or ulcers [82]. The clinical response observed was ascribed to increased production of TGFβ in the wounded area, a growth factor known to affect angiogenesis, inflammatory reaction, granulation tissue formation, ECM deposition, re-epithelization and remodeling, thus promoting the healing process [84]. More recently, metformin has also been tested in combination with non-pharmacological approaches such as photobiomodulation for the effect on wound healing in DM type 2 rats. Interestingly, systemic administration of metformin and photobiomodulation showed a synergistic impact on skin repair by increasing fibroblasts, with improved formation of granulation tissue, by inducing new blood vessels and by modulating the inflammation and proliferation steps of wound healing [85].

### 6.4. Dipeptidyl Peptidase 4 (DPP4) Inhibitors

DPP4, also known as CD26, is a transmembrane serine aminopeptidase cleaving N-terminal dipeptides when proline, hydroxyproline, and alanine are present. Its main recognized function is cleavage of incretins, mainly glucagon-like pepide 1 (GLP1) and glucose-dependent insulinotropic polypeptide (GIP), that are released at enteric level and control insulin release. By cleaving in particular the N-terminal dipeptide of GLP-1, DPP4 promptly inactivates the insulin-releasing activity of the peptide [86]. Interestingly, DPP4 is not specific for GLP1, but targets several different substrates including neuropeptides, stromal cell derived factor 1α (SDF1α), and high mobility group box 1 (HMGB1) [87]. DPP4 inhibitors prolong the insulin-secretagogue effect of GLP1 and, together with GLP1 analogs, are among the treatment options available to be added to the therapy regimen when the hemoglobin A1c target is not achieved after 3 months of metformin [88]. Their use is particularly advantageous since they induce low rates of hypoglycemia and are considered weight neutral (DPP4 inhibitors) or can even induce weight loss (GLP1 analogs) [89]. In addition, the large distribution of DPP4 and its pleiotropic effects make this molecule an attractive target for therapeutic intervention beyond the classical use of DPP4 inhibitors as antiglycemic agents [90].

DPP4 is in fact expressed also in epithelial and endothelial cells, keratinocytes, and in subsets of macrophages and hematopoietic progenitor cells [87]. In endothelial cells, GLP1 (or its analogs) increases endothelial NO synthase (eNOS) activation and NO production, shows antioxidant and anti-inflammatory properties [91], and stimulates angiogenesis by promoting cell proliferation and migration [92]. 

DPP4 is one of the accessory molecules of helper T cells and facilitates T cell activation [93]. It also interacts with caveolin-1 present in antigen T cells with ensuing activation of NF-kB and the inflammatory response [94]. DPP4 seems also to play a main role at the skin where major sources of the enzyme are epidermal keratinocytes and dermal fibroblasts. Its expression has been shown to vary during different phases of the wound repair process and, more specifically, the presence of high levels of DPP4 in diabetic wounds in mice is indicative of the persistence of an inflammatory status that impairs the healing process. Accordingly, DPP4 knockout mice display accelerated wound closure [95] and inhibition of DPP4 activity results in enhanced re-epithelialization of impaired wound healing in diabetic animals [96]. This finds support also in studies carried out in keratinocytes cultured in vitro where expression of DPP4 is increased when a wound is mechanically produced in the monolayer and inhibition of both expression and activity of the enzyme results in improved wound repair [97]. This effect involves increased keratinocyte migration, is mediated by activation of MMPs, and likely occurs through increased HMGB1, one of the substrates of DPP4, already reported to exert chemotactic effects [98] and to accelerate wound closure in keratinocytes and fibroblasts [99,100] as well as in diabetic animals [98]. In keratinocytes, DPP4 inhibition and the ensuing increased production of SDF1α, has also been associated with enhanced epithelial to mesenchymal transition that appears crucial for wound repair [101]. Increased SDF1α at the injury site has also been shown to restore normal progenitor cell recruitment [102], thus promoting angiogenesis [103].

A deeper knowledge of the involvement of DPP4 in reparative processes in wounded skin has prompted studies to investigate the potential for this target in the treatment of diabetic wounds. In excisional wound tissue of diabetic rats, it was shown that activation of GLP1 receptors reduced the formation of superoxide anions, ameliorated wound closure by reducing cytokines and inflammatory cells infiltration [104], and by inducing angiogenic activity through the increased expression of VEGF and HIF1α [105]. Besides stimulation of proliferation and migration of endothelial cells in the wounded area, GLP1 increased also endothelial precursor cells thus facilitating capillary tube formation and neovascularization [104,106]. Finally, GLP1 appears to intervene also in the remodeling phase of the healing process by stimulating TGFβ1 production that, through the increased proliferation of fibroblasts, synthesis of collagen, stimulation of MMP activity, and remodeling of the extracellular matrix, is crucial for the healing event [104]. The accelerated and improved wound healing following inhibition of DPP4 in a murine excisional wound model was characterized by increased vascularization but also by improved dermal thickness of the wounded area [107]. These effects however were correlated with increased SDF1α concentrations at the wound site and SDF1α-mediated recruitment of progenitor cells, all events impaired in diabetes [102].

Translation to clinical practice is substantiated by few, but significant, studies carried out in diabetic patients with chronic non-healing foot (or leg) ulcers. Inhibition of DPP4 significantly increased wound closure rate compared to placebo, with a good control of the appearance of ulcer-related adverse effects including infection, osteomyelitis, and cellulitis [108]. Notably, ulcer specimens from DPP4 inhibitor-treated patients showed enhanced HIF1α and VEGF expression, reduced oxygen radical species, and increased capillary density, indicative of anti-inflammatory and pro-angiogenic activity in response to treatment [108]. Similar efficacy of a DPP4 inhibitor was reported in a different clinical trial with significant improvement in the healing rate compared to placebo and increased SDF1α levels and epithelial mesenchymal transition in the injured site [101]. 

## 7. Conclusions

The concept of drug repurposing has grown in recent years, based on the attitude to exploit the knowledge we have on drugs and, at the same time, try to use them to their full potential. This is of course advantageous in terms of research and development of new molecules and the financial burden they imply. Diabetes-related impairment of wound healing and its most serious expression, i.e., the diabetic foot ulcer, offer the great opportunity to occur with a sequence of cellular and molecular events that are now well described and characterized, thus allowing pharmacological intervention in a precise manner. Several drugs have shown the ability to improve skin reparative processes and we have here briefly focused our interest on a few molecules (or classes of drugs) that appear particularly promising in this regard. The availability of clinical data for each drug (or class of drugs) is also reported to provide an idea of the feasibility of practical therapeutic use for each of them (Table 2). 

From the large body of evidence found in the literature, several weaknesses emerge as in the case of phenytoin that, although investigated in different clinical trials, provides data that carry a poor methodological approach. Very different is the story with statins. These drugs, when administered systemically, are endowed with adverse events and do not allow significant concentrations at the skin to be achieved. Hence, new formulations for optimized topical delivery are now receiving great attention. 

Drugs that are already used in the treatment of diabetes could represent the more obvious pharmacological intervention. In this sense, probably the most interesting seem to be DPP4 inhibitors. They have provided good results in preclinical studies, either in vitro and in vivo in animal models of diabetes, and also in clinical trials. If their efficacy in wound healing comes out to be true, data, not only from clinical trials, but more importantly from post-marketing studies should provide us information on whether the prevalence of impaired wound healing and/or the appearance of diabetic foot ulcers is reduced in diabetic populations treated with DPP4 inhibitors. In addition, differently from other drugs examined here, DPP4 inhibitors have been tested only after systemic administration, but not in topical formulations that, if feasible, may reveal even more encouraging results. Probably the effects of DPP4 inhibitors are multifactorial and although GLP1 has been described to exert several properties aimed to repair injured tissue, a main role for SDF1α and HMGB1, other important substrates of DPP4, have also been claimed, very likely all contributing with diverse roles to the improvement of the healing response. 

Finally, particularly promising appears the possibility to combine all these treatments with non-pharmacological innovative approaches, as already tested with metformin and lovastatin, to achieve improved results on the wound reparative events. 

## Figures and Tables

**Table 1:** Chemical formulas and structures of the four classes of drugs analyzed here, that display good efficacy in the treatment of impaired wound healing.

Name	Chemical Formula	Chemical Structure
Statins	C_24_H_36_O_5_	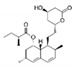
Phenytoin	C_15_H_11_N_2_NaO_2_	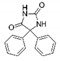
Metformin	C_4_H_11_N_5_	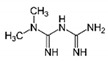
DPP-4 inhibitors	C_16_H_15_F_6_N_5_O	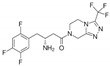

**Table 2:** List of drugs that display reparative effects in impaired wound healing.

Drugs	Effect on Wound Healing	Evidence from Human Studies	Administration
DPP-4 inhibitors	anti-inflammatory, anti-oxidant endothelial cell precursors proliferation-> angiogenesis fibroblast and keratinocyte migration wound remodeling	Improved healing of wounds and chronic foot ulcers in patients with diabetes [101,108].	systemic
metformin	anti-inflammatory, anti-oxidant endothelial cell precursors proliferation-> angiogenesis collagen deposition ECM organization	Improved healing in traumatic wound or ulcers [82].	topical
phenytoin	antibacterial, fibroblast proliferation-> granulation tissue increased VEGF release -> angiogenesis	Improved healing in a variety of wounds. Several randomized clinical trials available but methodologically poor. Reviewed in [64].	topical
statins	anti-inflammatory, angiogenesis	Mevastatin reverses several altered molecular pathways in ex vivo specimens derived from non healing edge of foot ulcers from diabetic patients [60,61].	topical
β-blockers	angiogenesis, proliferation of keratinocytes ECM organization	Only data in animals	topical
ACE-inhibitor	anti-oxidative	Only data in animals	topical
Estrogen (ERβ)	angiogenesis, proliferation of keratinocytes	Only data in animals	topical/systemic
